# Comparison of outcomes of laparoscopic sacrocolpopexy with concomitant supracervical hysterectomy or uterine preservation

**DOI:** 10.1007/s00192-023-05534-0

**Published:** 2023-04-13

**Authors:** Hirotaka Sato, Shota Otsuka, Hirokazu Abe, Sachiyuki Tsukada

**Affiliations:** 1 Department of Urology, Hokusuikai Kinen Hospital, Ibaraki, Japan; 2 Department of Urology, Kameda Medical Center, Chiba, Japan; 3 Department of Orthopedics, Hokusuikai Kinen Hospital, Ibaraki, Japan

**Keywords:** Pelvic organ prolapse, Preoperative, Prolapse, Recurrence, Risk factors, Sacrocolpopexy

## Abstract

**Introduction and hypothesis:**

Sacrocolpopexy was traditionally performed for post-hysterectomy prolapse or during concurrent hysterectomy. Sacrocolpopexy outcome with uterine preservation is poorly investigated. This study compared outcomes of laparoscopic sacrocolpopexy with concurrent supracervical hysterectomy or uterine preservation.

**Methods:**

This retrospective study compared data of patients with pelvic organ prolapse who underwent laparoscopic sacrocolpopexy with uterine preservation with the data of controls who underwent laparoscopic sacrocolpopexy with supracervical hysterectomy. We analyzed composite failure in uterine preservation versus concurrent supracervical hysterectomy (primary objective) and evaluated factors associated with the primary outcome of composite failure after laparoscopic sacrocolpopexy with preservation or supracervical hysterectomy (secondary objective). Composite failure was defined as subjective bulge symptoms, reoperation, or anatomical prolapse. Cox models indicated time to composite failure as an endpoint.

**Results:**

Of 274 patients, 232 underwent laparoscopic sacrocolpopexy with supracervical hysterectomy and 42 underwent laparoscopic uterine preservation. After propensity score matching (ratio: 2, for the laparoscopic sacrocolpopexy with supracervical hysterectomy group), 56 patients (24.1%) were in the supracervical hysterectomy group and 28 (66.7%) in the uterine preservation group. All patients underwent 24 months of follow-up. The composite failure rates were 10.7% for supracervical hysterectomy and 3.6% for preservation (*p*=0.87). The mean estimated blood loss was 10 ml (preservation, 10.0 ml [5.0–10.0] versus supracervical hysterectomy, 10.0 ml [10.0–15.0]; *p*=0.007). In the Cox proportional hazards model, higher preoperative body mass index and the point Ba increased composite failure risk.

**Conclusions:**

Although not statistically significant, composite failure in the two techniques is likely clinically meaningful.

**Supplementary information:**

The online version contains supplementary material available at 10.1007/s00192-023-05534-0.

## Introduction

Pelvic organ prolapse (POP) is a urogynecological disorder whereby the pelvic organs herniate into the vagina because of ligament or muscle weakness [1]. In Japan, 14,717 POP surgeries were performed in hospitals under the comprehensive payment system in 2020 [2]. Because of the large aging population, POP surgery rates and associated hysterectomies are expected to increase in Japan [3].

When surveyed about the POP surgery method, 36% of women indicated that they preferred uterine preservation (UP) if the outcome of the methods was the same [4]. Compared with hysterectomy, POP surgery with UP requires a shorter operative time (OT) and is associated with a lower estimated blood loss (EBL), superior perioperative outcomes, potentially lower risk of mesh exposure, and less invasive procedures. Additionally, the short-term outcome of the prolapse is unchanged [5]. Hence, there is increased interest in the role of UP during POP surgery [6–9].

We previously reported that using laparoscopic sacrocolpopexy (LSC) with UP for older or immunocompromised women [10] shortens the OT and decreases EBL. Here, a propensity score matching (PSM) analysis [11] was performed to compare LSC with UP (LSC/UP) and LSC with supracervical hysterectomy (SCH; LSC/SCH) techniques to reduce the effect of selection bias associated with both techniques.

There is a lack of evidence of UP, concomitant surgery, total hysterectomy, and SCH with LSC for women with a uterus [12]. Furthermore, no reports exist on the long-term outcomes of these two techniques in Japan.

The primary objective of this study was to analyze composite failure (CF) in the two groups at 24 months. The secondary objective was to evaluate factors (age, body mass index [BMI], the point Ba measurement, and anterior and posterior mesh implants) associated with the primary outcomes of CF after LSC/UP or LSC/SCH. To our knowledge, this is the first report to balance covariates within a population and compare the CF of the two techniques.

## Materials and methods

### Study design

This large case series was conducted by a surgeon (H.S.) who was also responsible for all patient follow-ups, in accordance with the Declaration of Helsinki for experiments involving humans. Study protocol approval and publication consent for this retrospective comparative study performed at Hokusuikai-Kinen Hospital were provided by the Institutional Review Board (approval number: 2022–077). The need for informed consent for participation was waived because of the retrospective study design. Clinical outcomes of all cases were assessed 24 months postoperatively.

### Data collection

The medical records of consecutive patients who underwent LSC/UP or LSC/SCH between 2 August 2015 and 1 February 2019 were reviewed. Patient demographic data, including age, BMI, and Charlson Comorbidity Index (CCI) [13], were collected. Additionally, we obtained the preoperative and postoperative POP Quantification (POP-Q) stage and perioperative data provided in the medical charts, such as OT, EBL, and whether single- or double-compartment mesh implantation was performed. The inclusion criteria were age ≥18 years, LSC/UP or LSC/SCH for preoperative POP-Q stage II or higher, and at least 24 months of follow-up. The exclusion criterion was a previous hysterectomy.

The POP stage was defined as the most severe stage in one or more anterior, apical, or posterior vaginal compartments. During follow-up, patients underwent a physical examination to diagnose anatomical failure (AF). This was performed by a single surgeon (H.S.) at 1, 3, 12, and 24 months and then annually thereafter. Symptoms were assessed using the Pelvic Floor Distress Inventory-20 (PFDI-20) [14]. Subscales of the PFDI included the POP Distress Inventory-6 (POPDI-6), Urinary Distress Inventory-6 (UDI-6), and Colorectal-Anal Distress Inventory-8 (CRADI-8). POP was examined using a split speculum while the patient performed the Valsalva maneuver. A urodynamic study was performed on women with urinary incontinence, symptoms of urinary urgency, and stress urinary incontinence (SUI). Postoperative AF indicating POP was defined as POP-Q stage II or higher in at least one compartment. CF was defined as the presence of one of the following: AF, which was further defined as Ba, Bp (the uppermost point of the posterior vaginal wall), or C, the lowest edge of the cervix ≥−1 (POP-Q stage II) in the supine lithotomy position during the 24-month assessment period; subjective failure (SF; presence of vaginal bulge symptoms), which was explained as an affirmative response to question 3 of the PFDI-20; and surgical re-treatment for prolapse.

### Surgical technique

All surgeries were conducted by a trained urologist (H.S.) following our operative procedure [10]. Briefly, either LSC/UP or LSC/SCH was selected based on the results of the preoperative consultation with the patients and considering their wishes after appropriate counseling. LSC/UP was recommended for patients who strongly desired UP, for older patients requiring less invasive procedures, and for those without uterine lesions. LSC was performed with anterior dissection to the level of the bladder neck and posterior dissection to the levator ani muscle. Two separate sheets of polypropylene mesh (Gynemesh; Ethicon, Somerville, NJ, USA) were sutured to the vagina using permanent non-absorbable sutures (Tefdesser II; Kono Seisakusyo, Chiba, Japan). In the UP group, the right broad ligament was opened at the cervico-uterine junction level in a vascular-free space outside the uterine artery and passed through the cephalic portion of the anterior mesh. Subsequently, if the posterior mesh was present, posterior dissection of the levator ani muscles was performed bilaterally, and the posterior mesh was fixed to the levator ani muscles. The anterior and posterior mesh pieces were then sutured together bilaterally to the uterine cervix. In the SCH group, anterior vaginal wall dissection was performed as in the LSC/UP group. If a posterior mesh was present, the anterior and posterior meshes were fixed bilaterally to the cervical stump and uterosacral ligaments. The peritoneum over the sacral promontory was incised, and the presacral area was dissected to expose the anterior longitudinal ligament. The sacral arm of the mesh was fixed to the anterior longitudinal ligament overlying the sacral promontory using permanent sutures. The peritoneum was sutured over the mesh using a 2–0 barbed suture (Stratafix spiral Monocryl plus a knotless tissue control device; Ethicon. Somerville, NJ, USA). For patients without posterior compartment prolapse (POP-Q stage ≥II), mesh implants were placed in the anterior vaginal compartment rather than the posterior compartment. For patients with colorectal symptoms and posterior compartment prolapse (POP-Q stage ≥2), laparoscopic posterior colporrhaphy [15] was concomitantly performed. Using the laparoscopic approach, the rectovaginal space was dissected until the levator ani muscle was visible. After identification of the rectovaginal fascia (RVF), a 2–0 barbed suture was passed from the perineal body to the right and left RVF and was tied. The RVF was completely closed using a continuous suture. Prophylactic antibiotics were administered within 60 min before surgical incision.

### Statistical analysis

Sample sizes of 56 and 28 patients in the LSC/SCH and LSC/UP groups respectively provided a post-hoc power of 45% to detect CF in the two groups (3.6% and 10.7% respectively), based on a 5% type I error rate. Variables included in the PSM were selected based on prior studies [16–20] and factors that may clinically influence outcomes. All covariates (potential confounders) affecting the outcomes were selected. Logistic regression analysis was performed to select control subjects whose propensity scores were “close” to those of the UP subjects. A matching method was used to calculate a population with covariates as similar as possible; only 28 of the 42 LSC/UP cases were selected. The only PSM diagnosis was an evaluation of the standardized mean differences criterion of <0.20.

A comparison between LSC/UP and LSC/SCH was performed using PSM (1:2) to evaluate CF. The UP and SCH cohorts were matched at baseline according to age [16], BMI [17], CCI, vaginal parity, POP-Q stage [18], follow-up, Ba measurement [19], and anterior or anterior and posterior mesh implants [20]. We used the Kaplan–Meier method and log-rank test to compare the CF rates of the matched groups. As the univariate analysis had many variables, we applied backward variable selection with *p* values <0.20. The stratified Cox proportional hazards model was used to account for the lack of independence of the PSM samples when estimating treatment effect variance and hazard ratios (HR; for LSC/UP versus LSC/SCH) with 95% confidence intervals (CI). Other between-group comparisons were performed using the Chi-squared test for categorical variables and Student’s *t* test for continuous variables. Continuous variables are expressed as mean ± standard deviation (SD) or median and interquartile range (IQR). Categorical variables are expressed as frequencies and percentages. All tests were two-sided with significance set at *p*<0.05. All statistical analyses were performed using R (R Foundation for Statistical Computing, Vienna, Austria) and EZR (Saitama Medical Center, Jichi Medical University, Saitama, Japan). We performed two sensitivity analyses: one with AF as the leading edge of any compartment beyond the hymen, and a Cox proportional hazards model to calculate HR and 95% CI.

## Results

**Fig. 1:**
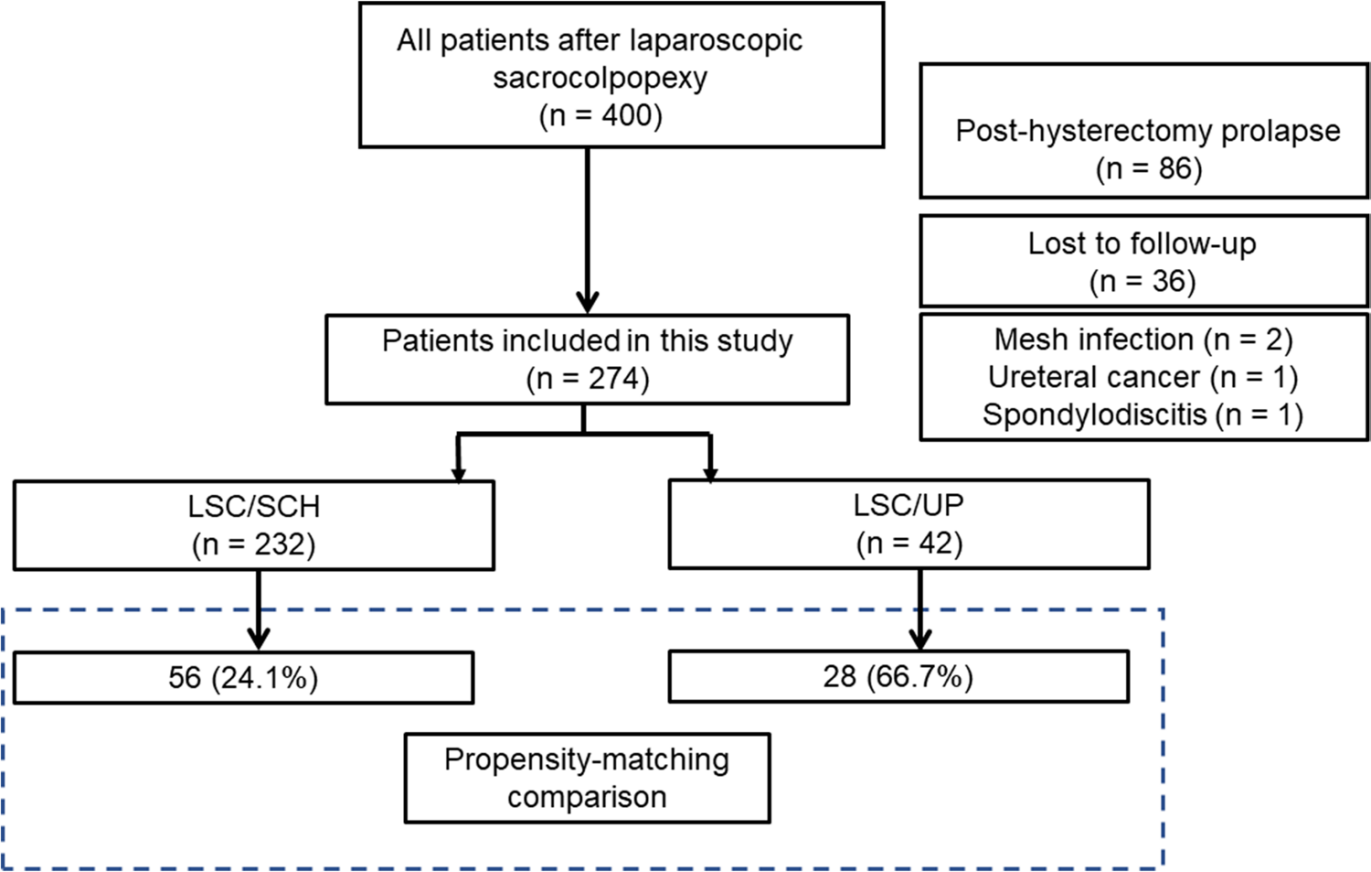
Patient distribution as per study design. *LSC/SCH* laparoscopic sacrocolpopexy/supracervical hysterectomy, *LSC/UP* laparoscopic sacrocolpopexy/uterine preservation

**Fig. 2:**
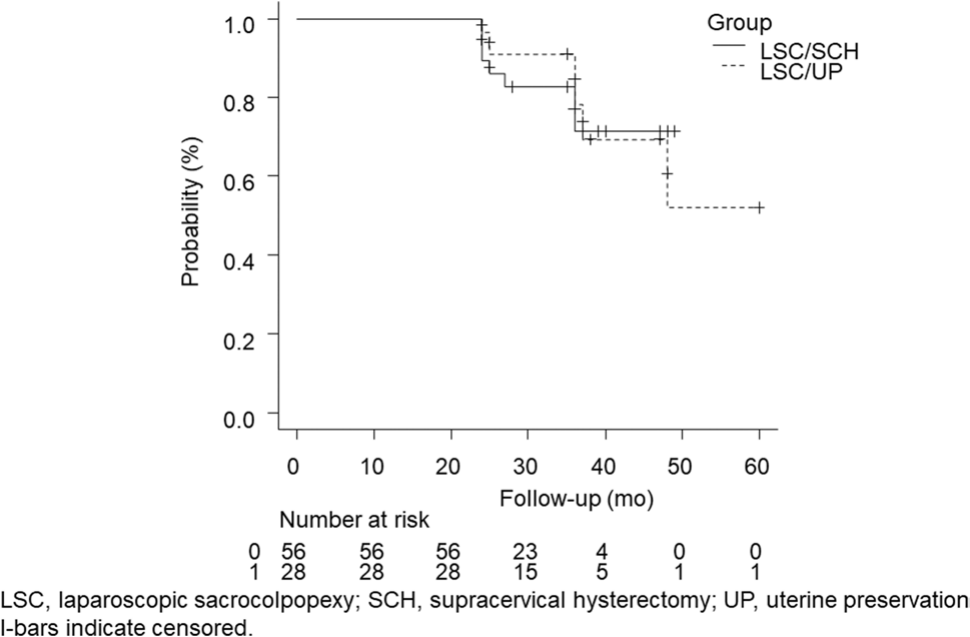
The Kaplan–Meier method and log-rank test to compare the composite failure rates of the matched groups. *LSC* laparoscopic sacrocolpopexy, *SCH* supracervical hysterectomy, *UP* uterine preservation. l-bars indicate censored

During the study period, 42 and 232 patients underwent LSC/UP and LSC/SCH respectively. After propensity scoring, the present analysis included 28 patients (66.7%) who underwent LSC/UP and 56 (24.1%) who underwent LSC/SCH (Fig. 1). The patients included in this study were subjected to PSM (1:2), with a control group of patients who underwent LSC/SCH. Table 1 summarizes the baseline demographic characteristics of the study groups; they were similar in both groups after PSM. Perioperative results showed that the OT durations of patients who underwent LSC/UP were similar to the OT durations of those who underwent LSC/SCH (133 [120–179] vs 143 [118–180] min; *p*=0.81), and that the mean EBL in both groups was 10 ml (LSC/UP group, 10.0 ml [5.0–10.0] vs LSC/SCH group, 10.0 ml [10.0–15.0]; *p*=0.007), which was not clinically significant (Table 2). Tables 3, 4 and Fig. 2 show the patients who met the definition of CF at 24 months. The outcomes were analyzed using the log-rank test. The CF rates were 10.7% (95% CI, 5.0–22.3) and 3.6% (95% CI, 0.5–22.8) for the LSC/SCH and LSC/UP groups respectively (*p*=0.87). Seventy-nine patients (94%) completed the PFDI-20 questionnaire 24 months postoperatively. The differences in the symptom scores from baseline to 24 months postoperatively of the two groups were compared (Tables 3, 4). The differences in the mean POPDI-6 scores for the LSC/SCH and LSC/UP groups were −24.8 and −23.9 points respectively; the corresponding differences for the mean CRADI-8 scores were −5.5 and −9.5 points respectively, and that for the mean UDI-6 scores they were −13.8 and −11.2 points respectively.

**Table 1:** The baseline demographic characteristics of the study groups

	LSC/SCH (*n*=232)	LSC/UP (*n*=42)	*p*	LSC/SCH (*n*=56)	LSC/UP (*n*=28)
	Before PSM			After PSM	
Age, years	70.3±7.3	73.6±6.2	0.006*	73.4±6.1	73.0±6.9
BMI, kg/m^2^	24.6±3.1	24.0±2.9	0.31	23.9±3.4	24.3±3.3
Parity number: 0 and 1	15 (6.5)	4 (9.5)	0.72	5 (8.9)	2 (7.1)
Parity number: 2	124 (53.4)	22 (52.4)		29 (51.8)	15 (53.6)
Parity number: 3 or more	93 (40.1)	16 (38.1)		22 (39.3)	11 (39.3)
CCI	0 (0–0)	1.0 (0–1.8)	<0.001*	0 (0–1.0)	1.0 (0–1.0)
Constipation	15 (6.5)	3 (7.1)	0.75	6 (10.7)	2 (7.1)
Former tobacco use	4 (1.7)	0 (0)	1.000	0 (0)	0 (0)
Before SUI	78 (35.1)	21 (50.0)	0.082	15 (28.8)	14 (51.9)
Before UUI	70 (31.5)	13 (31.0)	1.00	15 (28.8)	9 (33.3)
Follow-up, months	36.6±13.0	30.9±9.2	0.006*	29.8±7.6	33.3±10.1
Previous POP surgery	4 (1.7)	1 (2.4)	0.57	1 (1.8)	1 (3.6)
POP-Q stage II	15 (6.5)	2 (4.8)	0.95	3 (5.4)	1 (3.6)
POP-Q stage III	190 (81.9)	36 (85.7)		43 (76.8)	24 (85.7)
POP-Q stage IV	27 (11.6)	4 (9.5)		10 (17.9)	3 (10.7)
POP-Q point Ba, cm	1.2±2.5	2.4±2.0	0.004*	2.5±2.4	1.9±2.1
POP-Q point Bp, cm	−0.7±2.1	−0.3±2.0	0.25	−0.08±2.2	−0.54±2.0
POP-Q point C, cm	−0.5±3.9	−0.8±3.9	0.70	1.2±3.6	−1.4±4.2
POP-Q point TVL, cm	7.2±1.0	6.8±1.1	0.02*	6.9±0.9	7.0±1.3

These asterisks are statistically significant: *P* < 0.05

Data are shown as numbers (%), means (± SD), or medians (IQR)

*Ba* the superior-most location of the front vaginal wall, *Bp* the uppermost point of the posterior vaginal wall, *BMI* body mass index, *C* the lowest edge of the cervix, *CCI* Charlson Comorbidity Index, *IQR* interquartile range, *LSC* laparoscopic sacrocolpopexy, *POP* pelvic organ prolapse, *POP-Q* Pelvic Organ Prolapse Quantification, *PSM* propensity score matching, *SCH* supracervical hysterectomy, *SD* standard deviation, *SUI* stress urinary incontinence, *TVL* total vaginal length, *UP* uterine preservation, *UUI* urge urinary incontinence

These encompass factors that were included in the propensity score matching, age, BMI, parity, CCI, POP-Q stage, follow-up, Ba measurement, and anterior or anterior and posterior mesh implants

**Table 2:** Perioperative details

	Before PSM			After PSM		
Characteristic	LSC/SCH (*n*=232)	LSC/UP (*n*=42)	*p*	LSC/SCH (*n*=56)	LSC/UP (*n*=28)	*p*
Single mesh	87 (37.5)	23 (54.8)	0.041*	32 (57.1)	12 (42.9)	0.25
Double mesh	145 (62.5)	19 (45.2)		24 (42.9)	16 (57.1)	
OT, min	160 (130–190)	130 (110–169)	0.001*	133 (120–179)	143 (118–180)	0.81
EBL, ml	10.0 (10.0–20.0)	10.0 (5.0–10.0)	0.001*	10.0 (10.0–15.0)	10.0 (5.0–10.0)	0.007*
LOA	32 (13.8)	6 (14.3)	1.00	12 (21.4)	4 (14.3)	0.56
BSO	76 (32.8)	1 (2.4)	< 0.001*	16 (28.6)	1 (3.6)	0.008*
LPC	38 (16.4)	6 (14.3)	0.82	16 (28.6)	2 (7.1)	0.026*

Data are shown as numbers (%) or medians (IQR)

*BSO* bilateral salpingo-oophorectomy, *EBL* estimated blood loss, *IQR* interquartile range, *LOA* lysis of adhesions, *LPC* laparoscopic posterior colporrhaphy, *LSC* laparoscopic sacrocolpopexy, *OT* operative time, *PSM* propensity score matching, *SCH* supracervical hysterectomy, *UP* uterine preservation

**Table 3:** Patients meeting the composite failure definition at 24 months

	LSC/SCH (*n*=56) vs LSC/UP (*n*=28)				*p*
Characteristics	Percentage	95% CI	Percentage	95% CI	
Composite failure^a^	10.7	5.0–22.3	3.6	0.5–22.8	0.87
Anatomical failure	5.4	1.8–15.7	0	NA	0.99
Reoperation	3.6	0.9–13.5	0	NA	0.75
Anterior compartment prolapse	6.0	0.9–13.5	0	NA	0.31
Apical compartment prolapse	1.8	0.3–12.0	0	NA	0.92
Posterior compartment prolapse	1.8	0.3–12.0	0	NA	0.70
Subjective failure	5.7	1.9–16.5	3.8	0.6–24.3	0.91

^a^A composite failure was defined as at least one of the following: anatomical prolapse (Pelvic Organ Prolapse Quantification stage ≥2); bulging reported when completing the PFDI-20; or retreatment

*CI* confidence interval, *LSC* laparoscopic sacrocolpopexy, *NA* not applicable, *SCH* supracervical hysterectomy, *UP* uterine preservation

**Table 4:** Preoperative and postoperative symptoms scores and comparison of differences in the symptom scores at baseline and 24 months for those who underwent LSC/SCH or LSC/UP

	LSC/UP (*n*=28) versus LSC/SCH (*n*=56)						*p* for mean difference
	Pre	Post	Mean difference	Pre	Post	Mean difference	
PFDI-20 (/300)	73.6±48.6	33.0±27.9	−44.2±46.9	93.5±52.2	49.9±35.0	−44.6±65.0	0.98
POPDI-6 (/100)	31.9±23.4	7.6±10.8	−24.8±21.9	35.6±23.0	11.2±13.5	−23.9±27.8	0.87
CRADI-8 (/100)	15.2±13.6	9.6±8.5	−5.5±11.8	22.6±16.0	14.0±11.0	−9.5±16.4	0.22
UDI-6 (/100)	29.3±22.2	15.8±14.8	−13.8±25.5	35.4±21.7	24.7±16.8	−11.2±29.3	0.69

Data are shown means (±SD)

PFDI-20 data of five patients were missing

*CRADI* Colorectal-Anal Distress Inventory, *LSC* laparoscopic sacrocolpopexy, *PFDI* Pelvic Floor Distress Inventory, *POPDI* Pelvic Organ Prolapse Distress Inventory, *SCH* supracervical hysterectomy, *UP* uterine preservation, *UDI* Urinary Distress Inventory

Table 5 shows the predictive factors for CF according to the stratified Cox proportional hazards model (secondary objective). We performed a model adjusted for age, BMI, Ba measurement, and number of meshes. BMI and Ba had adjusted HR (adjHR) of 1.17 (95% CI 0.89–1.53; *p*=0.26) and 1.46 (95% CI, 0.76–2.79; *p*=0.25) respectively. Supplementary Table 1 shows the CF results with AF defined as the leading edge of any compartments beyond the hymen. Supplementary Table 2 presents the Cox proportional hazards model results regarding predictive factors.

**Table 5:** Factors associated with composite failure: a stratified Cox proportional hazards model of propensity score-matched patients who underwent LSC/SCH or LSC/UP

Characteristics	adjHR	95% CI	*p*
Age	1.04	0.87–1.25	0.66
BMI	1.17	0.89–1.53	0.26
Ba (per centimeter)	1.46	0.76–2.79	0.25
Double mesh, yes	0.25	0.042–1.50	0.13

*adj HR* adjusted hazard ratio, *Ba* the superior-most location of the front vaginal wall, *BMI* body mass index, *CI* confidence interval, *LSC* laparoscopic sacrocolpopexy, *SCH* supracervical hysterectomy, *UP* uterine preservation

## Discussion

In this study, the CF rates at 24 months were 10.7% and 3.6% for LSC/SCH and LSC/UP respectively. Regarding CF, LSC/SCH did not differ in AF compared with the LSC/UP group. Anterior compartment recurrence was observed in 6.0% of cases. In comparison, apical and posterior compartment recurrences were observed in 1.8% of cases in the LSC/SCH group, and there was also no AF in any compartment in the LSC/UP group. Similarly, the reoperation rate was only 3.6% in the LSC/SCH group. There was no statistically significant difference in these results between the groups at 24 months.

According to a comparative study of LSC/SCH and LSC/UP [7], the 2-year AF rates were 8.6%, 5.2%, and 0% for anterior, posterior, and apical compartment prolapse respectively in the LSC/SCH cases. In the LSC/UP group, the AF rates were 7.7%, 7.7%, and 5.1% for anterior, posterior, and apical compartment prolapse respectively. Gagyor et al. [8] reported that there was no difference in recurrent compartments between the LSC/UP and LSC/SCH groups regarding apical and posterior vaginal compartments at 1 year of AF. However, there was a significant difference in the anterior vaginal compartment between the LSC/UP (21.1%) and LSC/SCH (7.7%) groups (*P*=0.017) because the feasibility of our standardized technique regarding anterior compartment prolapse recurrence was not influenced by the choice of UP. As a cause of anterior compartment recurrence, LSC/UP is associated with a difficulty in inserting the mesh into the anterior vaginal wall.

We also found that with LSC/UP, as the cephalic mesh penetrated the right broad ligament just outside the right uterine artery and was suspended to the sacrum, the mesh extending from the site of penetration to the back of the broad ligament was no longer visible. This occurred during mesh tension adjustment under laparoscopic guidance; therefore, blind mesh adjustment may be the key to success.

We also noted a difference between the POPDI scores at baseline and 24 months; however, improved subjective outcomes were similar in the two groups. The minimal important difference (MID) of at least −21 points for the POPDI was reached in both groups [21]. The MID of at least −11 points for the UDI was similar in the two groups [22]. The decrease in the CRADI did not reach a MID of −11 in the two groups [23]. We observed that 25% and 7.1% of women in the LSC/SCH and LSC/UP groups (*p*=0.075) respectively had concomitant posterior repair. However, the LSC/SCH group had a smaller MID than did the LSC/UP group, suggesting that posterior repair might not be associated with colorectal symptom improvement [24].

Stress urinary incontinence was present in 51% of women (143 out of 274) who underwent LSC, 21 of whom required an additional mid-urethral sling for persistent SUI. Women with POP and concomitant SUI who underwent LSC benefited from a two-step approach [25], as only 15% required additional incontinence procedures.

The clinical risk factors affecting recurrence in both groups were examined using stratified Cox regression analyses; regardless of UP or SCH with LSC, an elevated Ba point did not reach statistical significance for recurrence (adjHR, 1.46; 95% CI, 0.76–2.79, *p*=0.25), but was clinically important. Two recent studies [7, 19] reported that a severe cystocele (Ba ≥3 cm) and a Ba point >2 cm were associated with recurrence, regardless of the procedure performed.

Higher BMI did not reach statistical significance for recurrence (adjHR, 1.17; CI, 0.89–1.53; *p*=0.26), but was clinically important. Obesity may indirectly or directly contribute to surgical recurrence and is associated with an increased EBL, prolonged OTs, and increased intraoperative complications, which may reduce surgery effectiveness [26].

Compared with hysterectomy, LSC/UP reduces the OT and risk of organ injuries [27] and is expected to decrease EBL [28]. In this study, the LSC/UP group did not have a clinically significant EBL compared with the LSC/SCH group. However, LSC/UP is preferable when LSC is necessary for older patients with complex comorbidities.

Uterine preservation with POP surgery is a risk factor for future surgery because of new uterine or ovarian abnormalities. The risk of developing premalignant and malignant gynecological pathological conditions when undergoing uterine prolapse surgery is estimated to be approximately 1–3% [29]. Therefore, patients with UP should be counseled on the need for ongoing surveillance following practice guidelines [7].

This study had some limitations. Residual confounding may persist in our analysis of the retrospectively collected data. Important variables, such as genital hiatus ≥4 cm [30] were unavailable. The assessors were not blinded, and the survey was conducted by the attending surgeon, which both reduce generalizability and internal validity. Nonetheless, having one surgeon perform all the surgeries enhanced reliability.

In conclusion, our results demonstrated no difference in CF between the two techniques, although there was a trend toward a meaningful difference; our study design was underpowered to detect the difference.

Women with obesity and increased Ba points may experience uterine prolapse recurrence, regardless of UP or SCH. LSC/UP may benefit older women and women with comorbidities.

## Supplementary information

Below is the link to the electronic supplementary material.

Supplementary file1 (PDF 154 KB)

## Data Availability

The datasets generated and/or analyzed during the present study are available from the corresponding author on reasonable request.
