# Supplementary material for: Comparison of outcomes of laparoscopic sacrocolpopexy with concomitant supracervical hysterectomy or uterine preservation
Source: Int Urogynecol J. 2023 Apr 13;34(9):2217–24. doi: 10.1007/s00192-023-05534-0 (PMC10506926; doi:10.1007/s00192-023-05534-0)
Supplement: Supplementary file 1 — Supplementary file1 (PDF 154 KB) [file 192_2023_5534_MOESM1_ESM.pdf]

Supplementary Table 1. Patients meeting the composite failure definition at 24 months and comparison of the differences in symptom scores at baseline and 24 months for LSC/SCH vs LSC/UP.

	LSC/SCH (n = 52) vs. LSC/UP (n = 26)				
Characteristic	(%)	95% CI	(%)	95% CI	*P*
Composite failure*	9.8	4.2–22.0	3.8	0.6-24.3	0.95
Anatomical failure (%)	3.9	1.0–14.8	0	NA	0.72
Reoperation	2.0	0.3–13.1	0	NA	0.29
Anterior compartment prolapse	2.0	0.3–13.1	0	NA	0.33
Apical compartment prolapse	2.0	0.3–13.1	0	NA	0.48
Posterior compartment	2.0	0.3–13.1	0	NA	0.66

prolapse					
Subjective failure	2.1	0.3–14.2	4.2	0.6–26.1	0.26

* Composite failure was defined as presence of at least one of the following: anatomical prolapse (leading edge of any compartment beyond the hymen), bulging reported when completing the PFDI-20, retreatment. PFDI-20 data of five patients were missing.

CI, confidence interval; CRADI, Colorectal-Anal Distress Inventory; LSC, laparoscopic sacrocolpopexy; MUS, mid-urethral sling; NA, not applicable; POPDI, Pelvic Organ Prolapse Distress Inventory-6; POP-Q, Pelvic Organ Prolapse Quantification; SCH, supracervical hysterectomy; UP, uterine preservatio

Supplementary Table 2. Factors associated with composite failure: Cox proportional hazards model of propensity score-matched patients who underwent LSC/SCH or LSC/UP.

Characteristic	Univariate analysis			Multivariate analysis		
	HR	95% CI	*P*	HR	95% CI	*P*
Age	1.02	0.93–1.11	0.68	1.005	0.89–1.13	0.94
Age: years ≥ 60	0.53	0.068–4.08	0.54			
BMI	1.11	0.97–1.26	0.13	1.23	1.04–1.45	0.01*
BMI ≥ 25	1.17	0.40–3.38	0.77			
Ba (per centimeter)	1.20	0.95–1.52	0.12	1.26	0.92–1.72	0.14
Ba ≥ 2 cm	2.13	0.57–8.00	0.26			
LSC/UP, yes	0.92	0.33–2.57	0.88			
Preoperative POP-Q stage IV	1.47	0.42–5.17	0.55			
Parity, reference number of 0 and 1						

Number of 2	1.58	0.20–12.46	0.66			
Number of 3 or more	1.10	0.13–9.57	0.93			
Double mesh, yes	0.17	0.053–0.52	0.0021*	0.18	0.033–0.99	0.049*
Previous POP surgery	4.12	0.90–19.0	0.069	0.80	0.16–10.57	0.80
LOA, yes	1.27	0.27–5.98	0.76			
Constipation, yes	0.95	0.12–7.3	0.96			

Ba, the most superior location of the front vaginal wall; BMI, body mass index; CI, confidence interval; LOA, lysis of adhesions; LSC, laparoscopic sacrocolpopexy; POP, pelvic organ prolapse; POP-Q, pelvic organ prolapse quantification; SCH, supracervical hysterectomy; UP, uterine preservation.
